# Differential expression of inflammatory responsive genes between chronic periodontitis and periodontally affected bronchiectasis patients

**DOI:** 10.22099/mbrc.2020.37397.1508

**Published:** 2020-12

**Authors:** Abhaya Gupta, Neetu Singh, Anil Kumar, Umesh Pratap Verma, Ajay Kumar Verma, Hari Shyam, Nand Lal, Surya Kant, Ankur Kumari

**Affiliations:** 1 Department of Periodontology, Faculty of Dental Sciences, King George’s Medical University, Uttar Pradesh, Lucknow, India; 2 Department of Molecular Biology, Center for Advanced Research, King George’s Medical University, Uttar Pradesh, Lucknow, India; 3 Department of Respiratory Medicine, King George’s Medical University, Uttar Pradesh, Lucknow, India

**Keywords:** Tumor necrosis factor superfamily, Lymphotoxin A gene, Bronchiectasis, Chronic periodontitis, Expression

## Abstract

The study aimed to investigate differential expression of targeted inflammatory-immune responsive genes *[LTA, LTB, TNFSF4, TNFSF11/RANKL, TNFSF13, TNFSF13B, TNFRSF11B/ Osteoprotegerin; OPG *and *GFPT1/GFA* ] in gingival tissues of bronchiectasis patients having chronic periodontitis in North central Indian population. Gingival tissues were collected from 30 systemically healthy chronic periodontitis patients (CP), 30 bronchiectasis patients with chronic periodontitis (B+CP), 3 systemically healthy with healthy gingiva (healthy control; HC) and 3 bronchiectasis with healthy gingiva (bronchiectasis control; BC). Statistical analysis revealed 7 genes to be significantly upregulated on comparing CP with B+CP i.e *LTA *(P<0.0001) in B+CP while *LTB* (P<0.0001), *TNFSF4* (P=0.0003), *TNFSF11* (P<0.0001), *TNFSF13* (P=0.0003), *TNFSF13B* (P<0.0001) and *TNFRSF11B* (P=0.0004) in CP group. *LTA (Lymphotoxin A)* gene could be a potential genetic marker in bronchiectasis patients with chronic periodontitis.

## INTRODUCTION

An emerging prevalence of bronchiectasis and periodontitis has been noticed worldwide. Recently, a systematic meta-analysis had validated association between chronic respiratory diseases (chronic obstructive pulmonary disease; COPD, asthma, pneumonia) and periodontitis [1]. Interestingly, bronchiectasis patients have demonstrated considerable overlapping of clinical phenotypes with COPD and asthma [2]. Although, direct evidence associating bronchiectasis and periodontitis is lacking, yet both diseases have progressive chronicity with immune-infective-inflammatory etiopathogenesis [3, 4]. Additionally, neutrophil priming and spillage of pro-inflammatory cytokines locally and systemically from lungs and periodontium could be plausible links. Expression of genes encoding tumor necrosis factor (TNF) and its receptor superfamilies’ (TNFSF and TNFRSF) have decisive role in autoimmune, respiratory, periodontal diseases etc. 

We hypothesized that pulmonary inflammation in bronchiectasis patients can bring about dysregulation in expression of immune-inflammatory genes in their gingival tissues if concurrently affected by chronic periodontitis. Therefore, the study aimed at analysing differential expression of relatively neglected inflammatory-immune responsive genes *LTA, LTB, TNFSF4, TNFSF11, TNFSF13, TNFSF13B, TNFRSF11B and GFPT1/GFA* in inflamed gingival tissues of bronchiectasis patients compared to systemically healthy chronic periodontitis counterparts. 

## MATERIALS AND METHODS

134 participants (age range 18-70 years) were screened out of which 66 fulfilled inclusion criteria. Bronchiectasis are cases with cough, sputum production, and/or recurrent pulmonary infections with dilated bronchi (HRCT thorax). Chronic periodontitis are those with pocket probing depth (PPD) ≥5mm, clinical attachment levels (CAL) ≥5mm and bleeding on probing. Healthy gingiva had a PPD≤3.0 mm, CAL≤3.5 mm, and no bleeding on probing.

Based on clinical and radiological parameters, participants grouped into chronic periodontitis (CP; n=30) were systemically healthy individuals with chronic periodontitis; bronchiectasis with chronic periodontitis group (B+CP; n=30) had presence of both diseases. Two control groups (n=3) i.e. healthy control (HC) who were systemically healthy with healthy gingiva and bronchiectasis control (BC) having bronchiectasis with healthy gingiva were included. 

Anaesthetized interproximal gingiva with no prior supra- or sub-gingival instrumentation was dissected (3mm^2^) and placed in RNA stabilizing agent *(RNA later)* immediately to be stored at -80°C until RNA isolation. Gene primers were designed using Fast PCR software (PrimerDigital Ltd.) (Table 1)

**Table 1:** Primer details of immune-inflammatory genes used in study

**Primers**	** Sequences**	**Product (bp)**	**Efficiency (%)**
*LTA* F *LTA* R *LTB* F *LTB* R *TNFSF11* F *TNFSF11* R *TNFRSF11B* F *TNFRSF11B* R *TNFSF13* F *TNFSF13* R *TNFSF13B* F *TNFSF13B* R *TNFSF4* F *TNFSF4* R *GFPT1* F *GFPT1* R *Beta Actin* F *Beta Actin* R	5' TCCCAAGGGTGTGTGGCACCAC 3' 5'AACCATCCTGGAGGAAGGCACG 3' 5'GGCGTTTCTGACGAGCGGGA 3' 5'GTCCAGCACTGGAGTCACCGTC 3' 5'ACCATGATCGGGGTTGGGCCA 3' 5'CGGATCCAGTAAGGAGGGGTTG 3' 5'ATTTCGCTCTGGGGTTCCAG 3' 5'TTGACGTACTGCAGCTCCTTGC 3' 5'CAACATGGGGGGCCCAGTCAGA 3' 5'CAGGCTTCCAGGGCATCGGA 3' 5'CTGCAACCTTGCTGCTGGCA 3' 5'TCTGGACCCTGAACGGCACG 3' 5'ACTTCCATGTGAATGGCGGAGA 3' 5'GGCAATCTTGGGGTGTGACG 3' 5'ATCCCCGAGGGAGTCGTGTC 3' 5'TTGCAGGCATTGGCTTCCCA 3' 5'TCA CCC ACA CTG TGC CCA TCT ACG A3' 5'CAG CGG AAC CGC TCA TTG CCA ATG G3'	249 241 341 301 234 271 314 296 295	99 98 99 99 98 99 98 97 98

**Note: **Forward Primer; F, Reverse Primer; R

Two samples in B+CP group showed degradation on agarose gel electrophoresis and hence excluded. TRI-Reagent (Invitrogen Life Technologies, CA) was used for RNA extraction from samples followed by cDNA synthesis (cDNA kit; Promega Corporation, USA) and gradient PCR run. Relative gene expression was quantified using 2^-ΔCt ^method compared house-keeping gene (Beta Actin) by qRT-PCR. Graph Pad Prism software (San Diego, CA) was used for statistical analysis. P value was calculated by Mann-Whitney U test with P<0.05 (significant); P<0.01 (moderately significant); P<0.001 (highly significant).

## RESULTS AND DISCUSSION

Demographics of participants presented in Table 2 whereas Table 3 depicts relative gene expression of targeted markers in various groups. Only *LTB* (P=0.0199) and *TNFSF13/APRIL* (P=0.0117) were significantly upregulated in CP patients compared to HC while rest displayed insignificant results. Comparison between BC and B+CP groups showed insignificant difference in all genes except *TNFSF4* (P=0.0487) and *TNFRSF11B/OPG* (P=0.0164) with significant upregulation in BC patients. However, between CP and B+CP, *LTB, TNFSF4, TNFSF11, TNFSF13, TNFSF13B* and *TNFRSF11B* genes were upregulated (high significance) in CP but interestingly, only *LTA* gene got significantly upregulated in B+CP ( P<0.0001).

**Table 2:** Demographics of participants

**Characteristics**	**HC (N=3)**	**CP (N=30)**	**BC (N=3)**	**B+CP (N=30)**
Age (years)^a^	55±15.39	35.8±11.6	41±23.64	41±14.3
Gender (Male/Female)	2/1	15/15	3/0	15/15
Radiographic type (Cylindrical/Cystic/Mixed)	N/A	N/A	0/3/0	5/15/8
% Lung side affected (Right/Left/Both lungs)	N/A	N/A	66.6/0/33.3	20/20/60
PPD (mm)^a^	1.00	6.01±0.74^b^^, c^	2.06±0.90	5.21±1.01^d^
CAL (mm) ^a^	1.33±0.57	6.69±0.83^e,^^f^	2.46±1.10	6.21±0.60^g^

^a ^Mean±SD; PPD: ^b^P=0.0109 in CP *vs* HC, ^c^P=0.0026 in CP *vs* B+CP, ^d^P=0.0422 in B+CP *vs* BC; CAL: ^e^P=0.004 in CP *vs* HC, ^f^P=0.02 in CP *vs* B+CP, ^g^P=0.03 in B+CP *vs* BC.

**Table 3:** Relative gingival tissue expression (fold change) of markers in various groups as determined by RT-PCR

**Inflammatory cytokines** ^a^	**HC** **(N=3)**	**CP** **(N=30)**	**P**			**BC** **(N=3)**	**B+CP** **(N=28)**	**P**		**CP** **(N=30)**	**B+CP (N=28)**	**P**	
**LTA**	1.02±0.20	1.9±1.02	0.106			1.02±0.22	2.23±1.14	0.076		0.96±0.49	2.76±1.42	<0.0001	
**LTB**	1.01±0.18	3.06±1.18	0.019			1.04±0.33	0.78±0.42	0.332		3.06±1.19	0.79±0.42	<0.0001	
**TNFSF4**	1.26±0.83	1.22±1.10	0.684			1.07±0.13	0.66±0.54	0.048		1.79±1.16	0.70±0.57	0.0003	
**TNFSF11/** **RANKL**	1.23±0.89	16.40±18.9	0.199			4.29±6.06	1.68±4.94	0.332		16.40±18.9	1.68±4.94	<0.0001	
**TNFSF13/** **APRIL**	1.06±0.39	20.19±19.1	0.011			1.01±0.08	5.87±7.74	0.150		20.20±19.1	5.87±7.75	0.0003	
**TNFSF13B**	1.12±0.57	25.94±29.9	0.125			0.12±0.15	0.19±0.34	0.712		25.90±29.9	0.19±0.34	<0.0001	
**Inflammatory cytokine receptor**														
**TNFRSF11B/OPG**	1.08±0.36	0.71±0.64	0.158			1.1±0.76	0.25±0.29	0.016		0.71±0.65	0.25±0.29	0.0004	
**Immune response marker**												
**GFPT1/GFA**	1.13±0.58	0.87±0.87	0.332			1.06±0.37	1.21±1.81	0.239		0.87±0.87	1.21±1.81	0.794	

^a ^Mean±SD, Mann-Whitney U test

Cystic radiological phenotype along with extensive lungs involvement was prevalent in our bronchiectasis patients thereby explaining high systemic inflammatory burden. Significant gingival upregulation of *LTA* gene in B+CP patients was seen despite lower clinical PPD and CAL values as compared to CP. It might be due to excessive outpouring of signalling molecules from bronchiectatic lungs and primed systemic neutrophils that upon reaching peripheral inflamed gingiva result in genetic variability. Studies have demonstrated *LTA *gene polymorphisms in periodontitis [5] and COPD [6] patients to be associated with enhanced susceptibility. To our knowledge, our study is the first one to quantify *LTA* gene expression in gingival tissues of chronic periodontitis as well as bronchiectasis with chronic periodontitis patients.

No studies are available showing role of *LTB* gene either in bronchiectasis or chronic periodontitis. While, *TNFSF4 *gene was found to be associated with chronic inflammatory pathways in both periodontal and pulmonary tissues. Upregulation of *RANKL* and downregulation of *TNFRSF 11B *gene is proven in periodontal diseases and it corroborate with our genetic and clinical periodontal findings also. *TNFSF13* and *TNFSF13B* have critical roles in B cell development and activation respectively. However, only one study utilizing microarray dataset has shown upregulation of *TNFSF13B* in chronic periodontitis subjects emphasizing its role as a potential prognostic biomarker [7]. So, there is paucity of literature either in relation to periodontitis or bronchiectasis or both that warrants future research.

Although our results in respect to aforementioned genes have favoured an inflammatory link by getting overexpressed at inflammatory periodontal sites i.e CP group. Somehow, they were downregulated in B+CP group despite local and pulmonary inflammation. Strikingly, inflammatory genes (blood cells and lung tissue) were found to be differentially expressed in exacerbators compared to those with zero exacerbations in COPD [8] with which bronchiectasis bears clinico-pathophysiological resemblance. Hence, exacerbations and some systemic immune dysregulation rather than exclusive pulmonary immune defect in chronic airway diseases (COPD, bronchiectasis etc.) could be responsible for this genetic variance.

In conclusion, *LTA* gene could be regarded as a novel genetic gingival biomarker for bronchiectasis whereas *LTB, TNFSF4, TNFSF11 (RANKL), TNFRSF 11B (OPG), TNFSF 13* and *TNFSF13B* for chronic periodontitis. Validation of these predicted disease relevant genes through large observational studies utilizing bioinformatics technologies with larger sample size is required to provide further pathophysiological insight of these chronic inflammatory diseases and in predicting disease risk. However, despite our limitations, this is a significant initial observation under expansion with more such studies.
